# Meta-Analysis of AI Integration in Abdominal Imaging for Liver Fibrosis and MASLD: Evaluating Diagnostic Accuracy and Clinical Impact

**DOI:** 10.3390/jcm14238466

**Published:** 2025-11-28

**Authors:** Rosa Alba Pugliesi, Karim Ben Mansour, Jonas Apitzsch, Angeliki Papachristodoulou, Vasileios Rafailidis, Douglas S. Katz

**Affiliations:** 1 Section of Radiology, Department of Biomedicine, Neuroscience and Advanced Diagnostics (BiND), University of Palermo, Via del Vespro 129, 90127 Palermo, Italy; 2 Department of Radiology, Hôpital de Morges, Chem. du Crêt 2, 1110 Morges, Switzerland; karim.ben.mansour@hotmail.com; 3 Department of Radiology and Nuclear Medicine, Helios Hospital Pforzheim, 75175 Pforzheim, Germany; jonas.apitzsch@helios-gesundheit.de; 4 Department of Radiology, Ahepa University Hospital Thessaloniki, St. Kiriakidi 1, 54636 Thessaloniki, Greece; 5 Department of Radiology, King’s College Hospital, Denmark Hill, London SE5 9RS, UK; 6 Department of Radiology, NYU Langone Hospital–Long Island, 259 First Street, New York, NY 11501, USA

**Keywords:** artificial intelligence, liver fibrosis, metabolic dysfunction-associated steatotic liver disease (MASLD), diagnostic accuracy, abdominal imaging

## Abstract

**Background/Objectives**: To evaluate the diagnostic accuracy of artificial intelligence (AI)-based imaging techniques for liver fibrosis and metabolic dysfunction-associated steatotic liver disease (MASLD). **Materials and Methods:** We performed a comprehensive search in PubMed, Embase, Cochrane Library, and Web of Science until August 2025. A total of 15 studies (mean age of patients 56 years, 60% male) were included. The risk of bias in the included studies was assessed using the Quality Assessment of Diagnostic Accuracy Studies 2 (QUADAS-2) tool. Diagnostic performance metrics were calculated using a random-effects bivariate model, including the area under the curve (AUC), sensitivity, specificity, positive and negative likelihood ratios, and diagnostic odds ratio. Meta-regression analysis was conducted to investigate potential sources of heterogeneity when I^2^ was ≥50%. A *p*-value < 0.05 was considered statistically significant. **Results:** For liver fibrosis, pooled sensitivity was 0.85, specificity was 0.81, and AUC was 0.92. For MASLD, sensitivity was 0.86, specificity was 0.95, and AUC was 0.99. Different imaging modalities and AI classifiers caused significant study heterogeneity. To avoid misleading pooled estimates across varied datasets, imaging modality and AI model subgroup analyses were performed. Only three studies were used to estimate MASLD; therefore, considerable between-study heterogeneity should be considered. **Conclusions:** AI-based imaging modalities demonstrate promising diagnostic accuracy for liver fibrosis and MASLD, warranting further standardization to enhance diagnostic consistency.

## 1. Introduction

AI has made substantial strides in diagnostic radiology, particularly in the evaluation of liver conditions, including liver fibrosis and metabolic dysfunction-associated steatotic liver disease (MASLD) [1]. Traditional cross-sectional imaging techniques, including computed tomography (CT), magnetic resonance imaging (MRI), and ultrasonography, have been enhanced by AI technologies [2,3,4]. These advancements involve convolutional neural networks (CNNs) for analyzing complex imaging patterns, support vector machines (SVMs) for image classification, and deep learning models such as Residual Networks (ResNet) for improving feature extraction and classification accuracy [5]. The application of AI in this context focuses on qualitative assessments (e.g., presence or absence of disease) and semi-quantitative grading of liver fibrosis (mild, moderate, severe), as well as fat fraction imaging in MRI [6]. AI technologies are designed to automate image analysis, detect subtle patterns that may be missed by human reviewers, and integrate diverse data sources to enhance overall diagnostic performance [7].

Accurate diagnosis and staging of liver fibrosis and MASLD are crucial for effective patient management and therapeutic interventions [8]. The goals of utilizing AI are to provide reliable, consistent, and timely assessments that can support or even surpass traditional imaging methods [9,10,11].

Importantly, the nomenclature of fatty liver disease has recently changed: in June 2023 an international multi-society Delphi consensus renamed the formerly used term nonalcoholic fatty liver disease (NAFLD) to MASLD, and nonalcoholic steatohepatitis (NASH) to metabolic dysfunction-associated steatohepatitis (MASH) [12]. This change reflects increasing recognition of the central role of metabolic dysfunction in driving hepatic steatosis and seeks to provide positive diagnostic criteria rather than exclusion-based definitions [13]. Because the volume of literature published under the new MASLD definition remains modest, especially in imaging and AI integration, it is timely to update reviews and meta-analyses using the updated terminology and to clarify how prior work under the NAFLD heading maps to MASLD.

This meta-analysis evaluates the diagnostic performance of AI-integrated abdominal imaging modalities for liver fibrosis and NAFLD, aiming to assess the effectiveness of various AI techniques in detecting and staging these conditions and synthesizing current research findings.

## 2. Materials and Methods

This systematic review and meta-analysis followed the PRISMA guidelines [14] as described in Supplementary Table S1. This review protocol is registered with PROSPERO, with the registration number CRD42024592089.

### 2.1. Inclusion and Exclusion Criteria

Relevant articles were screened by title and abstract after removing duplicates. Studies were eligible for inclusion if they assessed the diagnostic performance of AI-based abdominal imaging modalities for liver fibrosis and metabolic dysfunction-associated steatotic liver disease (MASLD). Because the MASLD terminology was only introduced in 2023, studies published before this date generally referred to “non-alcoholic fatty liver disease (NAFLD)” while describing essentially the same disease spectrum. Therefore, for consistency and inclusivity, both NAFLD- and MASLD-related studies were considered, acknowledging their clinical and pathophysiological overlap. The remaining studies were then examined in full text to confirm eligibility. Inclusion criteria for articles were: (1) observational studies reporting the diagnostic performance of AI-based abdominal imaging modalities in the diagnosis or staging of liver fibrosis and/or MASLD (previously referred to as NAFLD); (2) articles had to specify the reference standard (diagnostic method(s)) and class(es) of AI; (3) publications reporting sensitivity and specificity data or providing sufficient information to calculate them; (4) articles that clearly reported training and test datasets or contained information on validation methods; and (5) studies published as original articles. Exclusion criteria were: (1) no full text electronically available; (2) publication in a language other than English; (3) comments, letters, editorials, protocols, guidelines, and review papers; (4) studies with insufficient outcome data; and (5) animal studies.

### 2.2. Search Strategy

We conducted a comprehensive literature search in PubMed, the Web of Science, Scopus, the Cochrane Library, Embase, Google Scholar, and CINAHL until 30 August 2025. Search terms included combinations of “artificial intelligence,” “deep learning,” “machine learning,” “imaging,” “liver fibrosis,” “steatosis,” “non-alcoholic fatty liver disease (NAFLD),” and “metabolic dysfunction-associated steatotic liver disease (MASLD)” to ensure inclusion of studies published both before and after the nomenclature change. We also manually searched the references mentioned in narrative reviews and pertinent non-systematic papers to find further relevant studies that our search approach could have overlooked. The detailed search strategy is described in Supplementary Table S2.

### 2.3. Screening and Selection Process

Two independent reviewers, K.B.M and A.P., both with over five years of experience in abdominal imaging and AI-based diagnostics, conducted the search and selection process. Each reviewer handled distinct sets of studies to ensure exhaustive coverage and accuracy. The kappa coefficient (k) was used to evaluate the inter-rater reliability for the review process and data extraction [15].

Discrepancies were resolved through discussion, ensuring consistent and precise study inclusion by J.A. (with 10 years of experience in radiology).

### 2.4. Data Extraction

The two independent authors conducted the data extraction process in duplicate. They retrieved information from the eligible articles following the predefined inclusion and exclusion criteria. Information was systematically collected on a standardized data sheet, which included the following variables: (1) study ID, (2) country, (3) study design (retrospective, cohort, prospective), (4) diagnosis (liver fibrosis or MASLD/NAFLD), (5) fibrosis stage (F1–F4), (6) abdominal imaging modality (MRI, CT, ultrasound, shear wave elastography), (7) total number of patients, (8) total number of images, (9) AI classifier (SVM, CNN, etc.), (10) AI performance metrics, (11) type of dataset (training, validation, or test), (12) sensitivity (%), (13) specificity (%), and (14) accuracy (%). Given that most included studies predated the 2023 MASLD terminology, diagnostic definitions were interpreted according to their equivalence to the current MASLD criteria, as described in the EASL–EASD–EASO Clinical Practice Guidelines [12]. Any inconsistencies in the extracted data were resolved by consulting a third reviewer with substantial expertise in diagnostic imaging. The accuracy of the data extraction process was ensured through systematic checks and cross-verification. Although the κ coefficient demonstrated almost perfect agreement, a total of three discrepancies occurred during screening, all of which were resolved by consensus. No formal calibration exercise was performed prior to screening.

### 2.5. Quality Assessment of the Studies

The methodological quality of the included studies was independently evaluated using the QUADAS-2 tool, which includes four criteria: “patient selection”, “index test”, “reference standard”, and “flow and timing”, and judges bias and applicability [16]. Each article was assessed in terms of risk of bias, and the first 3 domains were assessed with respect to applicability. Each item is answered with “yes,” “no,” or “unclear”. The answer of “yes” means low risk of bias, whereas “no” or “unclear” means the opposite, as attached in Supplementary Table S3. The Grading of Recommendations, Assessment, Development and Evaluations (GRADE) assessment tool was not used due to the challenge of applying it to diagnostic test accuracy (DTA) reviews [17]. The quality assessment was conducted by the two independent reviewers. Any discrepancy was settled through mutual discussion and consensus with the third reviewer. The results were presented using Review Manager 5.4.

### 2.6. Data Syntheses and Analyses

This diagnostic meta-analysis was conducted on the analytical software Meta-disk 1.4 and the statistical software Comprehensive Meta-Analysis version 3 (Biostat Inc., Englewood, CO, USA) in order to analyze the pooled sensitivity, specificity, Positive Likelihood Ratio (PLR), Negative Likelihood Ratio (NLR), Diagnostic Odds Ratio (DOR), and AUC values with 95% confidence intervals (CIs) across studies. As the terminology shift from NAFLD to MASLD occurred recently, the pooled analysis integrated both terms under a unified MASLD framework, reflecting the current understanding that both refer to the same metabolic liver disease spectrum. The data were considered statistically significant when two-sided *p* < 0.05. A random effects model was used in all analyses owing to an expectation of heterogeneity of data across studies [18]. The summary receiver operating characteristic (SROC) curve was also used based on the sensitivity and specificity of each study to assess the diagnostic performance [19].

Because of the differences in the basic features of the included articles, their diverging results may have been caused by heterogeneity or random errors. Therefore, the Cochrane chi-squared test was used to evaluate heterogeneity among articles, with *p* < 0.05 indicating the existence of heterogeneity [20]. To estimate the impact of heterogeneity on the meta-analysis, the I^2^ value was also calculated. If *p* < 0.05 and I^2^ > 50%, heterogeneity was defined as statistically significant [21]. In order to explore heterogeneity, the threshold effect was assessed using the Spearman correlation coefficient [22]. A strong positive correlation would suggest a threshold effect. Subgroup and random effects meta-regression analyses were also performed to identify potential sources of heterogeneity according to the type of abdominal imaging modality used and the AI classifier to identify which approaches seem to be most promising. We did not evaluate further covariates, such as age and sex, because they were not mentioned in all included papers. Also, there was no significant difference in study quality and sample size between studies, so they were excluded from subgroup and meta-regression analysis. Furthermore, a sensitivity analysis was conducted to evaluate the validity and robustness of the meta-analysis. Finally, Egger’s test was conducted to evaluate publication bias [23]. The latter was further assessed by the visual inspection of the symmetry in funnel plots.

## 3. Results

### 3.1. Study Selection

A total of 824 studies were initially identified through comprehensive database searches. Following the screening of 446 abstracts, 15 studies met the eligibility criteria and were included in the final systematic review and meta-analysis. The Kappa coefficient was 0.94, proving that the level of agreement between both authors was almost perfect.

**Figure 1:**
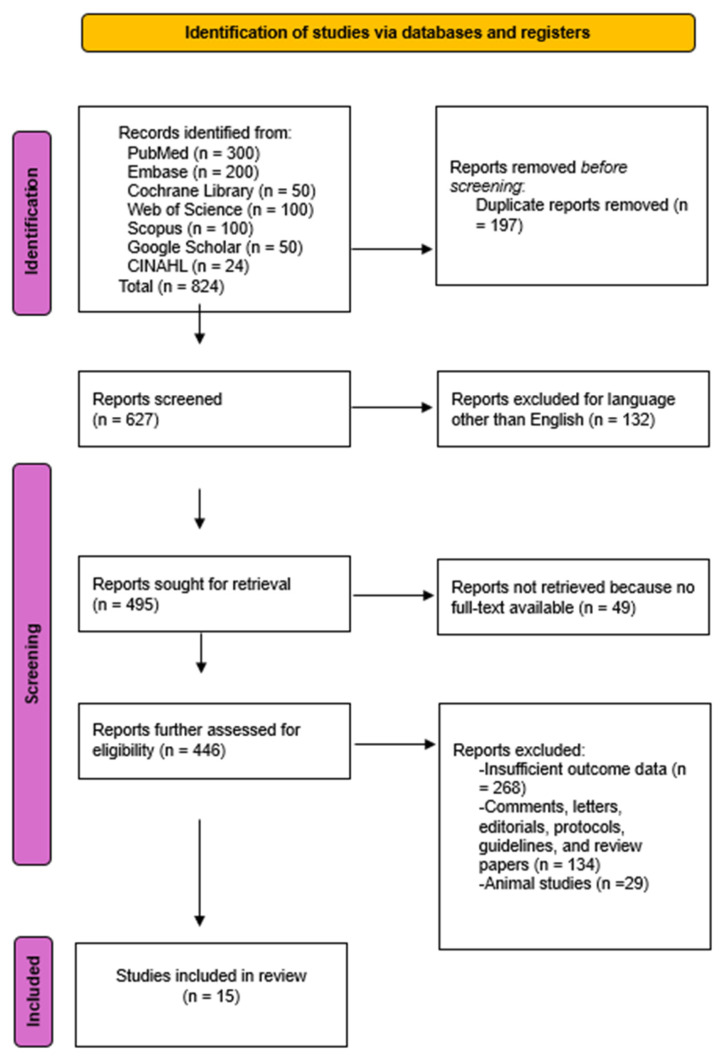
PRISMA Flow diagram of the literature study process and selection.

The PRISMA flowchart in Figure 1 details this process.

Although the updated MASLD terminology was included in our search, most eligible studies still referred to NAFLD, reflecting that the majority of AI-based imaging literature predates the 2023 redefinition. These studies, however, address the same metabolic liver disease continuum now classified as MASLD, supporting their inclusion in this analysis. Characteristics of included studies are summarized in Table 1.

### 3.2. Study Characteristics

The 15 studies assessed AI-based imaging for liver fibrosis and MASLD (previously NAFLD), published between 2012 and 2024 [24,25,26,27,28,29,30,31,32,33,34,35,36,37,38], across seven countries: China (*n* = M6), Republic of Korea (*n* = 2), USA (*n* = 2), Japan (*n* = 2), The Netherlands (n = 1), Egypt (*n* = 1), and Iran (*n* = 1). Study designs included retrospective (*n* = 7), prospective *(n* = 3), and cohort (*n* = 5) studies. Fibrosis staging definitions varied across studies, most commonly using the Kleiner system (F0–F4) [39].

The imaging modalities used were MRI (*n* = 5), CT (*n* = 4), ultrasonography (*n* = 4), shear wave elastography (*n* = 1), and transient elastography (*n* = 1). AI classifiers included CNNs, SVMs, ResNet, ANNs, Random Forest, and deep learning radiomics. Study sample sizes ranged from 37 to 7461 participants, with sensitivities from 63 to 97% and specificities from 59–100%.

To align pre-2023 NAFLD studies within the MASLD framework, only studies in which at least one metabolic risk factor was explicitly reported (obesity, diabetes, insulin resistance, dyslipidemia, or hypertension) were retained. Sensitivity analysis excluding two studies without clear metabolic profiling did not materially change pooled fibrosis estimates (ΔAUC < 0.02, Δsensitivity < 0.03), supporting the validity of this reclassification approach. When interpreted under the MASLD framework, these findings collectively represent AI’s growing potential to quantify hepatic steatosis and fibrosis across the metabolic disease spectrum.

The primary diagnostic effect size for this meta-analysis was the diagnostic odds ratio (DOR), with AUC and sensitivity/specificity treated as secondary accuracy metrics. Prediction intervals were additionally calculated for pooled DOR and AUC.

The AI classifiers included:

Convolutional Neural Networks (CNNs, *n* = 8): A type of deep learning model designed to automatically and adaptively learn spatial hierarchies of features from images. CNNs are particularly effective in image classification tasks [40].

Support Vector Machines (SVMs, *n* = 3): A machine learning algorithm that finds the optimal hyperplane to separate different classes in a dataset. It is effective for classification problems with a clear margin of separation [41].

ResNet (*n* = 1): A deep learning architecture that uses residual connections to help train very deep networks by mitigating the vanishing gradient problem [42].

Deep Learning Radiomics (*n* = 1): An approach combining deep learning with radiomics to extract and analyze features from medical images for improved diagnostic accuracy [43].

Artificial Neural Networks (ANNs, *n* = 1): A class of machine learning models inspired by the human brain, consisting of interconnected nodes (neurons) that process information in layers to learn complex patterns [44].

Random Forest (*n* = 1): An ensemble learning method that uses multiple decision trees to improve classification accuracy by averaging the results of individual trees to reduce overfitting and increase predictive performance [45].

Study sample sizes ranged from 37 to 7461 participants. Sensitivities varied from 63% to 97.2%, specificities from 59% to 100%, and accuracies from 74% to 98.64%. Disease stages reported included various fibrosis stages, i.e., ≥F2 (≥fibrosis stage 2), F1–F3 (fibrosis stages 1 through 3), and F0–F4 (fibrosis stages 0 through 4). AI classifiers were assessed through different methodologies, including cross-validation techniques, feature selection, and deep learning architectures, which reflect a broad range of approaches to enhancing diagnostic precision. More details about the methodological characteristics of the selected studies utilizing AI in abdominal imaging for liver disease, such as model types, validation strategies, and clinical implications, can be found in Supplementary Tables S4 and S5. The Kappa coefficient was 0.92, proving that the level of agreement between both authors was almost perfect.

### 3.3. Assessment of Risk of Bias

The quality of the 15 studies was methodologically assessed using the QUADAS-2 tool. With respect to domain patient selection, 4/15 studies were identified to have a high risk of bias because they did not use a consecutive or random sample. Interestingly, 9 studies showed a high risk of bias regarding the field of the index test due to not presetting the threshold. However, the domains of reference standard, flow, and timing were not affected by the risk of bias. In contrast, there were no concerns as to the applicability of the majority of studies included in this meta-analysis. Indeed, high applicability concerns were shown in 2 studies in patient selection and 7 studies in the index test. Tabular presentation for QUADAS-2 results of individual studies is attached as Supplementary Tables S6–S8.

### 3.4. Publication Bias

To evaluate the potential presence of publication bias, we employed a funnel plot asymmetry test, supplemented by Egger’s regression test. This dual-method approach ensures a thorough assessment of whether small-study effects or selective reporting biases might have distorted our meta-analytic findings. A funnel plot and Egger’s regression analysis identified a statistically significant publication bias (*p* = 0.000) for the DOR values associated with AI-based imaging for liver fibrosis. Visual inspection of the funnel plot (Figure S1) confirmed notable asymmetry, indicating the presence of publication bias. Because only three MASLD studies were available, publication bias testing and meaningful pooling are statistically unreliable and must be interpreted as exploratory only.

### 3.5. Findings

#### 3.5.1. Liver Fibrosis

**Figure 2:**
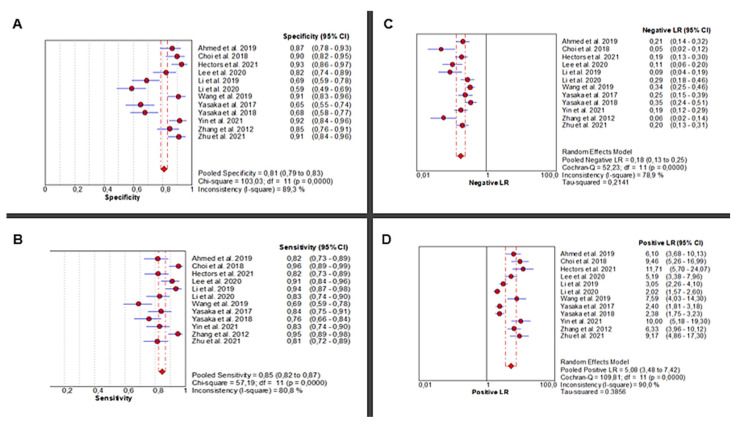
Diagnostic Performance of AI-Based Abdominal Imaging for Liver Fibrosis: Meta-Analysis Forest Plots. Forest plots display pooled specificity (**A**), sensitivity (**B**), negative likelihood ratio (**C**), and positive likelihood ratio (**D**) across 12 studies. Red circles show study estimates, blue lines their 95% CIs, red diamonds the pooled effects, red dashed lines their positions, and grey dashed lines reference values.

Twelve studies [24,25,26,27,28,29,30,31,32,33,34,35] investigated the diagnostic accuracy of AI-based abdominal imaging for the detection of liver fibrosis. The analysis demonstrated significant heterogeneity in sensitivity (Chi^2^ = 57.19, *p* < 0.0001; I^2^ = 80.8%), requiring a random-effects model for the pooled estimate. Sensitivity scores ranged from moderate to high and yielded a pooled estimate of 0.85 (95% CI: 0.82–0.87), representing excellent diagnostic performance. Specificity also showed high heterogeneity across studies (Chi^2^ = 103.03, *p* < 0.0001; I^2^ = 89.3%), with a pooled estimate of 0.81 (95% CI: 0.79–0.83) (Figure 2A,B).

The pooled positive likelihood ratio (PLR) demonstrated extreme heterogeneity (Chi^2^ = 109.81, *p* < 0.0001; I^2^ = 90%), with a pooled estimate of 5.08 (95% CI: 3.48–7.42), indicating a strong ability to correctly identify diseased individuals. Similarly, the negative likelihood ratio (NLR) was highly heterogeneous (Chi^2^ = 52.23, *p* < 0.0001; I^2^ = 78.9%), with a pooled value of 0.18 (95% CI: 0.13–0.25), supporting the use of AI to reliably rule out disease in healthy individuals (Figure 2C,D).

**Figure 3:**
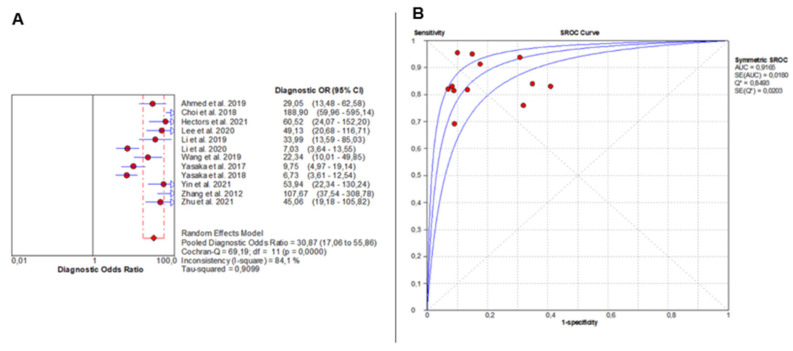
Overall Diagnostic Accuracy of AI-Based Imaging for Liver Fibrosis. The forest plot (**A**) shows diagnostic odds ratios across 12 studies, with red circles as study estimates, blue lines as 95% CIs, red diamonds as pooled effects, red dashed lines marking pooled positions, and grey dashed lines reference values. The SROC curve (**B**) uses red circles and blue curves, confirming high accuracy (AUC = 0.92). Detailed ranges of PLR, NLR, and DOR for individual studies are presented in Supplementary Table S9.

DOR analysis also showed significant heterogeneity (Chi^2^ = 69.19, *p* < 0.0001; I^2^ = 84.1%), with individual study estimates varying widely. The pooled DOR was 30.87 (95% CI: 17.06–55.86), indicating outstanding overall diagnostic accuracy (Figure 3A). The SROC curve yielded an AUC of 0.9165, confirming excellent diagnostic discrimination of liver fibrosis by AI-based imaging modalities (Figure 3B).

In the context of metabolic dysfunction-associated steatotic liver disease (MASLD), these findings underscore the critical role of AI in detecting and staging fibrosis, which represents the main prognostic determinant of disease progression. Although the majority of included studies predated the formal adoption of the MASLD terminology, the same metabolic and histopathological processes underline what was historically referred to as NAFLD. Thus, these results can be confidently interpreted within the MASLD framework, highlighting AI’s ability to support early, non-invasive risk stratification and improve longitudinal monitoring of hepatic fibrosis in metabolically driven liver disease.

#### 3.5.2. MASLD (Previously NAFLD)

**Figure 4:**
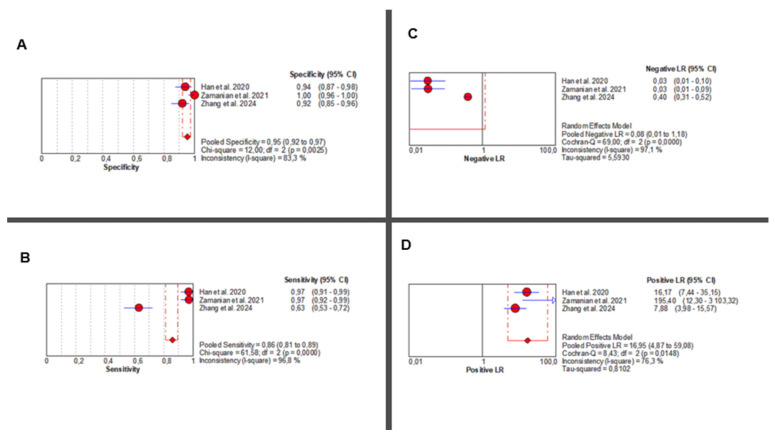
AI-Based Imaging for MASLD Diagnosis: Summary of Diagnostic Accuracy Metrics. Forest plots show pooled specificity (**A**), sensitivity (**B**), NLR (**C**), and PLR (**D**) from three studies. Red circles depict study estimates, blue lines their 95% CIs, red diamonds pooled effects, red dashed lines pooled positions, and grey dashed lines reference values. Results indicate high diagnostic performance with heterogeneity in likelihood ratios. The pooled positive likelihood ratio (PLR) was 16.95 (95% CI: 4.87–59.08), with significant heterogeneity (Chi^2^ = 8.43, *p* = 0.014; I^2^ = 76.3%). Negative likelihood ratio (NLR) results showed even greater inconsistency (Chi^2^ = 69.00, *p* < 0.001; I^2^ = 97.1%), with a pooled estimate of 0.08 (95% CI: 0.01–1.18) (Figure 4C,D). Corresponding individual study values are detailed in Supplementary Table S10.

Three studies [36,37,38] evaluated the diagnostic accuracy of AI-augmented abdominal imaging for MASLD. Given the limited number of included studies and considerable methodological heterogeneity, these pooled results should be interpreted with caution. Sensitivity was extremely heterogeneous (Chi^2^ = 61.58, *p* < 0.001; I^2^ = 96.8%), with a pooled estimate of 0.86 (95% CI: 0.81–0.89). Specificity was also highly variable (Chi^2^ = 12.00, *p* = 0.002; I^2^ = 83.3%), with a pooled estimate of 0.95 (95% CI: 0.92–0.97) (Figure 4A,B).

**Figure 5:**
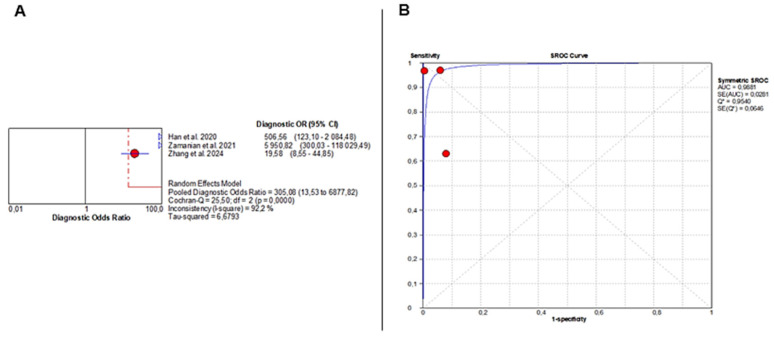
Diagnostic Accuracy of AI-Based Imaging for MASLD Detection. (**A**) Forest plot displays diagnostic odds ratios from three studies, with red circles indicating individual estimates, blue diamonds pooled effects, blue triangles study weights, and red and grey dashed lines marking confidence and reference intervals. (**B**) SROC curve shows excellent performance (AUC = 0.99), with red points representing study sensitivity–specificity pairs and the blue line depicting the fitted curve. Despite this high AUC, the extreme heterogeneity in sensitivity and specificity severely limits interpretability and precludes drawing definitive conclusions. The wide confidence intervals of several performance metrics further highlight the uncertainty surrounding the diagnostic accuracy of AI for MASLD.

The pooled DOR was 305.08 (95% CI: 13.53–6877.82), indicating very strong diagnostic performance but again with substantial heterogeneity (Chi^2^ = 25.50, *p* < 0.001; I^2^ = 92.2%) (Figure 5A). The SROC curve demonstrated near-perfect discrimination, with an AUC of 0.9881 (Figure 5B).

To assess whether variability in diagnostic thresholds contributed to the observed heterogeneity, a threshold effect analysis was conducted using the Moses model and inverse variance weighting. The Spearman correlation coefficient was 0.143 (*p* = 0.760), indicating no significant threshold effect across studies. Accordingly, the pooled diagnostic accuracy metrics are unlikely to be biased by threshold variation.

Given the very small number of included studies and the extreme between-study heterogeneity (I^2^ > 90% for several parameters), these pooled estimates are statistically unstable, and formal meta-analytic pooling may not be valid under these conditions. Therefore, the quantitative results should be interpreted cautiously and primarily as hypothesis-generating rather than confirmatory. A narrative interpretation is more appropriate for the MASLD subgroup until a larger number of methodologically comparable studies becomes available.

### 3.6. Subgroup Analyses

Subgroup analyses revealed variations in diagnostic accuracy depending on the imaging modality and AI classifier used. Meta-regression identified study quality, AI classifier type, and imaging modality as significant sources of heterogeneity. Statistically significant heterogeneity (*p* < 0.05) was observed across diagnostic performance metrics (sensitivity, specificity, PLR, NLR, DOR, and AUC). Further subgroup and meta-regression analyses for liver fibrosis studies showed no statistically significant differences between imaging modalities (*p* = 0.6962) or AI classifiers (*p* = 0.5479) (Table 2).

Additionally, to further reveal the likely origin of heterogeneity, a leave-one-out sensitivity analysis was performed. We revealed that DOR values of AI-based abdominal imaging modalities for the diagnosis of liver fibrosis did not differ markedly, which indicated that the meta-analysis had strong reliability. Indeed, the DOR values ranged from 19.431 (95% CI: 15.214–24.818), *p* = 0.000, to 26.225 (95% CI: 20.237–33.985), *p* = 0.000 (Table 3).

Sensitivity analysis could not be performed for MASLD due to the limited number of studies.

## 4. Discussion

This meta-analysis emphasizes the transformative potential of AI in the restructuring of the diagnostic landscape of liver disease, particularly within the contemporary framework of MASLD. The transition from the NAFLD terminology to MASLD is not merely a change in nomenclature; it is a paradigm shift that acknowledges the metabolic underpinnings of hepatic steatosis and fibrosis [46]. AI is a potent ally in this changing clinical context, capable of overcoming long-standing limitations in non-invasive liver imaging, including operator dependency, interpretative variability, and the limited sensitivity of traditional tools in early disease detection [47,48]. By integrating data from various imaging modalities, including CT, elastography, MRI, and ultrasound, AI exhibits a distinctive ability to standardize interpretation across centers and harmonize diagnostic procedures [49,50]. The complex spectrum of metabolic, hepatic, and cardiovascular interactions that characterize MASLD is contingent upon these characteristics [46]. Unlike traditional diagnostic scores, such as the FIB-4 or the NAFLD fibrosis score, which rely on indirect biochemical parameters [51], AI-driven imaging models derive complex, multidimensional data from raw imaging signals [52]. This capability enables the more comprehensive characterization of tissue texture, elasticity, and composition, thereby establishing a data-rich, objective, and reproducible framework for disease assessment [53].

The present findings support this evolving clinical role, as AI demonstrated high pooled diagnostic accuracy for liver fibrosis (sensitivity 0.85; AUC 0.92); however, prediction intervals indicated wide expected variability (DOR PI 3.9–172.4), underscoring that real-world performance may differ substantially from the pooled averages. This variability is consistent with the substantial heterogeneity (I^2^ > 80%) observed across studies, which was predominantly driven by differences in imaging modality, AI model architecture, fibrosis thresholds, reference standards, and study design.

Interpretation of pooled diagnostic accuracy requires caution because the included AI systems differed substantially in model architecture (e.g., CNNs, hybrid networks, radiomics-based approaches), imaging modalities (ultrasound, CT, MRI), and training datasets. Such methodological variability limits direct comparability across studies and reduces the stability of pooled metrics. Future research should implement standardized reporting guidelines, harmonized validation frameworks, and external multi-center testing to enable more reliable cross-study comparisons. The significance of AI in the diagnosis of MASLD is not limited to accuracy metrics from a clinical perspective. The genuine clinical value of this test is its capacity to identify subclinical or early fibrotic changes that frequently precede irreversible liver injury [54]. The most recent EASL–EASD–EASO Clinical Practice Guidelines [12] underscore the importance of detecting fibrosis progression prior to the onset of cirrhosis in the management of MASLD. In order to enable more personalized and timely interventions, these guidelines promote the development of improved non-invasive diagnostic instruments. The potential of AI to systematize quantification and risk stratification directly supports this agenda, as it offers scalable solutions that can be integrated into population-level screening programs [55]. Machine learning algorithms, including convolutional neural networks (CNNs), ResNet models, and support vector machines (SVMs), can identify intricate visual features that are invisible to the human eye [56]. These algorithms convert subjective interpretation into a standardized computational process [42,45,57]. This is particularly advantageous for MASLD, as it may be feasible to distinguish benign steatosis from early fibrotic transformation by making use of nuanced imaging distinctions.

However, the MASLD findings of this review must be interpreted cautiously. Only three studies were eligible for MASLD analysis, and extreme heterogeneity (I^2^ > 90%) produced unstable pooled estimates (AUC PI 0.71–1.00). Therefore, these results should be considered exploratory and hypothesis-generating rather than definitive. A narrative synthesis is more appropriate for this limited evidence base, as individual study effect sizes varied markedly and confidence intervals were wide.

When comparing endpoints, MASLD studies showed a pooled sensitivity of 0.86 and an AUC of 0.99, but the uncertainty was considerably higher than for fibrosis, limiting generalizability despite seemingly high point estimates.

Quantitatively, AI-based imaging demonstrated stronger and more consistent performance for liver fibrosis (pooled sensitivity 0.85, specificity 0.88, AUC 0.92) compared with MASLD, where the pooled AUC remained high (0.99), but estimates were unstable due to extreme heterogeneity (I^2^ > 90%). Fibrosis results were supported by narrower confidence intervals and a more reliable evidence base, whereas MASLD findings should be considered exploratory. The redefinition of NAFLD to MASLD does not invalidate prior research; rather, it contextualizes it within a broader metabolic spectrum. According to certain authors [58], the overlap between NAFLD and MASLD exceeds 95%, thereby guaranteeing that historical data remains highly pertinent. In fact, the current analysis is one of the most comprehensive and early attempts to reinterpret the existing AI-imaging literature through the MASLD lens. This alignment bolsters the study’s scientific contribution and novelty by illustrating how established datasets can inform the new nomenclature, thereby advancing toward a unified, metabolism-focused understanding of liver disease [59].

To ensure methodological rigor, earlier NAFLD studies were only reclassified as MASLD when metabolic dysfunction was explicitly documented, and sensitivity analyses excluding borderline cases produced no meaningful changes to pooled fibrosis accuracy (ΔAUC < 0.02), supporting the robustness of this approach. From a methodological perspective, the heterogeneity of clinical practice is reflected in the diversity of AI architectures and imaging modalities in this meta-analysis [60]. Although this diversity introduces variability, it emphasizes the real-world applicability and highlights the robustness of AI, whose consistent performance across diverse contexts is essential for clinical translation [61]. Separate meta-analyses by fibrosis thresholds (≥F2, ≥F3, F4) were not feasible because individual studies used heterogeneous staging cutoffs and did not consistently report threshold-specific accuracy metrics. Nevertheless, the interpretability of AI systems continues to be a significant obstacle. Black-box algorithms may generate precise predictions without furnishing clinicians with transparent reasoning [62]. The next generation of AI tools must integrate explainable AI (XAI) principles to establish trust and regulatory adoption. This involves linking model outputs to clinically interpretable imaging biomarkers and facilitating decision support, rather than opaque classification [63].

Risk-of-bias assessment revealed key methodological shortcomings across studies, particularly non-prespecified index test thresholds and mixed reference standards, which may inflate diagnostic accuracy and should be acknowledged when interpreting pooled estimates. In particular, several studies demonstrated great concern in key QUADAS-2 domains, most notably in the index test domain, where non-prespecified or data-driven thresholds increased the risk of inflated accuracy estimates. Additional concerns were identified in the reference standard and flow/timing domains in some studies, further underscoring the need for more rigorous methodological standardization in future AI-imaging research. An additional aspect of the impact of this work is its implications for global health equity. Populations with metabolic risk factors, including obesity and type 2 diabetes, are disproportionately affected by MASLD due to their global prevalence [64]. AI-driven imaging platforms have the potential to democratize access to dependable diagnostics in resource-limited environments where biopsy or specialized imaging expertise is unavailable [65]. The capacity of AI to seamlessly integrate with standard imaging devices, such as ultrasound, provides a practicable approach to broader implementation without the need for extensive infrastructural investment. This aligns with the multidisciplinary European guidelines [12], which emphasize the use of diagnostic strategies that are both cost-effective and scalable for the management of MASLD. Nevertheless, it is advisable to exercise caution as the field advances toward clinical deployment. Model performance can be influenced by algorithmic bias, variability in imaging quality, and inconsistent ground-truth definitions [66]. Transparent reporting, external validation, and multicenter standardization should be prioritized in future prospective studies to ensure reproducibility. Furthermore, the integration of clinical, biochemical, and imaging data through multimodal AI systems may lead to even more precise diagnostic signatures, thereby providing a comprehensive representation of the metabolic–liver interface [67]. This study demonstrates that AI is not simply a marginal enhancement to traditional imaging; instead, it is a transformative approach that augments the MASLD framework. Personalized hepatology is established by the amalgamation of clinical necessities and technology, guaranteeing objectivity, scalability, and equity. This meta-analysis sets a conceptual and methodological standard for future research by consolidating a varied range of data into the unified MASLD classification. The findings corroborate the claim that AI-driven imaging is poised to become a vital element of modern hepatology. Ultimately, technology will enhance patient outcomes by increasing the accuracy of care, facilitating earlier issue detection, and improving consistency.

## 5. Conclusions

AI-assisted imaging has substantially enhanced the diagnosis and treatment of metabolic dysfunction-associated steatotic liver disease. However, results for MASLD are based on only three heterogeneous studies and should be interpreted as preliminary. Through the integration of advanced computational algorithms and multimodal imaging, AI offers evaluations that are highly interpretable, reproducible, and standardized, surpassing those of conventional instruments. Future work must incorporate standardized validation frameworks, threshold harmonization, and external testing across imaging platforms before clinical deployment.

## Figures and Tables

**Table 1:** Summary of Key Studies on AI Classifiers for Liver Conditions: Study Details and Performance Metrics.

Study/Year	Journal	Country	Study Design	Imaging Modality	Stage	Total Patients	AI Classifier	Type of Set	Sensitivity	Specificity	Accuracy
Ahmed et al., 2019	NMR in Biomedicine	Egypt	Prospective	MRI	F1–F4	37	SVM	Validation	81.8%	86.6%	83.7%
Choi et al., 2018	Radiology	Republic of Korea	Retrospective	CT	≥F2	7461	CNNs	Test	95.5%	89.9%	94.1%
Han et al., 2020	Radiology	USA	Prospective	MRI	NS	204	CNNs	Test	97%	94%	96%
Hectors et al., 2021	Eur Radiol	USA	Retrospective	MRI	≥F2	355	CNNs	Test	82%	93%	85%
Lee et al., 2020	Eur Radiol	Republic of Korea	Retrospective	Ultrasonography	≥F2	3446	DCNN	External Test	91.3%	82.4%	86.6%
Li et al., 2019	Eur Radiol	China	Prospective	Ultrasound	≥F2	144	SVMs	Validation	93.8%	69.2%	81.5%
Li et al., 2020	Int J CARS	China	Retrospective	CT	≥F2	347	ResNet	Test	83%	59%	74%
Wang et al., 2019	Gut	China	Prospective	SWE	≥F2	398	CNN	Validation	69.1%	90.9%	81%
Yasaka et al., 2017	Radiology	Japan	Retrospective	MRI	≥F2	634	DCNN	Test	84%	65%	79%
Yasaka et al., 2018	Eur Radiol	Japan	Retrospective	CT	≥F2	286	DCNN	Test	74%	76%	75%
Yin et al., 2021	Eur Radiol	Netherlands	Retrospective	CT	≥F2	252	CNN	Test	83.0%	91.7%	88.3%
Zamanian et al., 2021	J Biomed Phys Eng	Iran	Prospective	Ultrasonography	NS	55	SVMs	Test	97.2%	100%	98.64%
Zhang et al., 2012	BMC Med Inform Decis Mak	China	Prospective	Duplex US	F1–F3	239	ANNs	Validation	95.0%	85.0%	88.3%
Zhang et al., 2024	JMIR Form Res	China	Prospective	TE	NS	916	Random Forest	Test	62%	90%	84%
Zhu et al., 2021	Contrast Media and Molecular Imaging	China	Prospective	MRI	F0–F4	123	CNN	Test	81.45%	91.12%	88.13%

Key details from studies on AI classifiers for liver conditions, including study ID, journal, country, design, diagnosis, imaging modality, sample size, AI technology, and performance metrics (sensitivity, specificity, accuracy). Abbreviations: CNN: Convolutional Neural Network. SVM: Support Vector Machine. ResNet: Residual Network. ANN: Artificial Neural Network. DCNN: Deep Convolutional Neural Network. NS: Not specified. F1–F4: Fibrosis stages from mild (F1) to advanced (F4). SWE: Shear Wave Elastography. TE: Transient Elastography.

**Table 2:** Subgroup and Meta-Regression Analysis of Diagnostic Accuracy in Liver Fibrosis Studies Based on Imaging Modality and AI Classifier.

AI-Based Abdominal Imaging Modality	Sensitivity (95%CI)	Specificity (95%CI)	PLR (95%CI)	NLR (95%CI)	DOR (95%CI)	AUC
Overall	0.85 (0.82–0.87)	0.81 (0.79–0.83)	5.08 (3.48–7.42)	0.18 (0.13–0.25)	30.87 (17.06–55.86)	0.9165
Imaging modality MRI CT Ultrasonography Shear wave elastography *p* = 0.6962	0.82 (0.78–0.86) 0.84 (0.80–0.88) 0.93 (0.90–0.96) NA	0.84 (0.80–0.87) 0.77 (0.73–0.81) 0.79 (0.74–0.83) NA	6.10 (2.53–14.70) 4.41 (1.99–9.77) 4.52 (2.81–7.30) NA	0.21 (0.17–0.26) 0.19 (0.10–0.37) 0.09 (0.06–0.13) NA	28.59 (12.46–65.57) 24.74 (6.03–101.52) 53.86 (28.78–100.79) NA	0.8958 0.9147 0.9628 NA
AI classifier CNN SVM Resnet Radiomics of elastography ANN *p* = 0.5479	0.85 (0.82–0.87) 0.88 (0.82–0.92) NA NA NA	0.83 (0.80–0.86) 0.78 (0.72–0.83) NA NA NA	5.80 (3.27–10.30) 4.19 (2.07–8.49) NA NA NA	0.18 (0.13–0.26) 0.15 (0.06–0.34) NA NA NA	34.82 (14.55–83.33) 30.99 (17.20–55.82) NA NA NA	0.9226 0.9037 NA NA NA

Subgroup and meta-regression analysis for liver fibrosis studies show variations in diagnostic accuracy based on imaging modality and AI classifier. No significant differences between subgroups indicate the reliability and versatility of AI-based diagnostics in clinical settings. NA: not applicable due to the limited number of studies. CNN: Convolutional Neural Network, SVM: Support Vector Machine, ResNet: Residual Network, ANN: Artificial Neural Network. PLR: Positive Likelihood Ratio. CI: Confidence Interval. NLR: Negative Likelihood Ratio. DOR: Diagnostic Odds Ratio. AUC: Area Under the Curve.

**Table 3:** Sensitivity Analysis of Diagnostic Odds Ratios for AI-Enhanced Imaging in Liver Fibrosis Diagnosis.

Study Excluded	DOR (95% CI)
Ahmed et al., 2019	20,763 (16,140–26,709) (*p* = 0.000)
Choi et al., 2018	19,431 (15,214–24,818) (*p* = 0.000)
Hectors et al., 2021	19,904 (15,535–25,500) (*p* = 0.000)
Lee et al., 2020	20,029 (15,614–25,692) (*p* = 0.000)
Li et al., 2019	20,742 (16,188–26,577) (*p* = 0.000)
Li et al., 2020	25,440 (19,675–32,892) (*p* = 0.000)
Wang et al., 2019	21,367 (16,629–27,454) (*p* = 0.000)
Yasaka et al., 2017	24,034 (18,606–31,044) (*p* = 0.000)
Yasaka et al., 2018	26,225 (20,237–33,985) (*p* = 0.000)
Yin et al., 2021	53.88 (44.75–64.57) (*p* = 0.000)
Zhang et al., 2012	19,934 (15,546–25,561) (*p* = 0.000)
Zhu et al., 2021	20,137 (15,694–25,837) (*p* = 0.000)

Sensitivity analysis of DOR values for AI-based imaging in liver fibrosis diagnosis. The analysis confirms stable DOR values with minimal variation, supporting the consistency and reliability of AI-based diagnostics across various study designs and populations.

## Data Availability

The extracted dataset is available in FigShare at: Pugliesi, Rosa Alba (2025). Meta-Analysis of AI Integration in Abdominal Imaging for Liver Fibrosis and MASLD: Evaluating Diagnostic Accuracy and Clinical Impact. figshare. Dataset. https://doi.org/10.6084/m9.figshare.30505748.

## Supplementary Materials

The following supporting information can be downloaded at: https://www.mdpi.com/article/10.3390/jcm14238466/s1, Table S1: PRISMA-DTA checklist. Table S2: Full database search strategies. Table S3: QUADAS-2 data extraction template. Table S4: Methodological characteristics of included studies. Table S5: Clinical implications and limitations of AI models. Table S6: QUADAS-2 assessments for all included studies. Table S7: Reviewer agreement for QUADAS-2 domains. Table S8: Summary of risk of bias and applicability across studies. Table S9: Diagnostic performance metrics for liver fibrosis studies. Table S10: Diagnostic odds ratios for MASLD studies. Figure S1: Funnel plot for pooled DOR analysis.
