# Supplementary material for: Meta-Analysis of AI Integration in Abdominal Imaging for Liver Fibrosis and MASLD: Evaluating Diagnostic Accuracy and Clinical Impact
Source: J Clin Med. 2025 Nov 28;14(23):8466. doi: 10.3390/jcm14238466 (PMC12693291; doi:10.3390/jcm14238466)
Supplement: Supplementary file 1 [file jcm-14-08466-s001.zip › jcm-3991168-supplementary.pdf]

Table S1:

Section/topic	#	PRISMA-DTA Checklist Item	Reported on page #
**TITLE / ABSTRACT**			
Title	1	Identify the report as a systematic review (+/- meta-analysis) of diagnostic test accuracy (DTA) studies.	1
Abstract	2	Abstract: See PRISMA-DTA for abstracts.	1
**INTRODUCTION**			
Rationale	3	Describe the rationale for the review in the context of what is already known.	1
Clinical role of index test	D1	State the scientific and clinical background, including the intended use and clinical role of the index test, and if applicable, the rationale for minimally acceptable test accuracy (or minimum difference in accuracy for comparative design).	2
Objectives	4	Provide an explicit statement of question(s) being addressed in terms of participants, index test(s), and target condition(s).	2
**METHODS**			
Protocol and registration	5	Indicate if a review protocol exists, if and where it can be accessed (e.g., Web address), and, if available, provide registration information including registration number.	2
Eligibility criteria	6	Specify study characteristics (participants, setting, index test(s), reference standard(s), target condition(s), and study design) and report characteristics (e.g., years considered, language, publication status) used as criteria for eligibility, giving rationale.	2
Information sources	7	Describe all information sources (e.g., databases with dates of coverage, contact with study authors to identify additional studies) in the search and date last searched.	2
Search	8	Present full search strategies for all electronic databases and other sources searched, including any limits used, such that they could be repeated.	3
Study selection	9	State the process for selecting studies (i.e., screening, eligibility, included in systematic review, and, if applicable, included in the meta-analysis).	3
Data collection process	10	Describe method of data extraction from reports (e.g., piloted forms, independently, in duplicate) and any processes for obtaining and confirming data from investigators.	3

Section/topic	#	PRISMA-DTA Checklist Item	Reported on page #

Definitions for data extraction	11	Provide definitions used in data extraction and classifications of target condition(s), index test(s), reference standard(s) and other characteristics (e.g. study design, clinical setting).	3
Risk of bias and applicability	12	Describe methods used for assessing risk of bias in individual studies and concerns regarding the applicability to the review question.	3
Diagnostic accuracy measures	13	State the principal diagnostic accuracy measure(s) reported (e.g. sensitivity, specificity) and state the unit of assessment (e.g. per-patient, per-lesion).	3-4
Synthesis of results	14	Describe methods of handling data, combining results of studies and describing variability between studies. This could include, but is not limited to: a) handling of multiple definitions of target condition. b) handling of multiple thresholds of test positivity, c) handling multiple index test readers, d) handling of indeterminate test results, e) grouping and comparing tests, f) handling of different reference standards	4
Meta-analysis	D2	Report the statistical methods used for meta-analyses, if performed.	4
Additional analyses	16	Describe methods of additional analyses (e.g., sensitivity or subgroup analyses, meta-regression), if done, indicating which were pre-specified.	4
**RESULTS**			
Study selection	17	Provide numbers of studies screened, assessed for eligibility, included in the review (and included in meta-analysis, if applicable) with reasons for exclusions at each stage, ideally with a flow diagram.	5
Study characteristics	18	For each included study provide citations and present key characteristics including: a) participant characteristics (presentation, prior testing), b) clinical setting, c) study design, d) target condition definition, e) index test, f) reference standard, g) sample size, h) funding sources	6
Risk of bias and applicability	19	Present evaluation of risk of bias and concerns regarding applicability for each study.	6-7
Results of individual studies	20	For each analysis in each study (e.g. unique combination of index test, reference standard, and positivity threshold) report 2x2 data (TP, FP, FN, TN) with estimates of diagnostic accuracy and confidence intervals, ideally with a forest or receiver operator characteristic (ROC) plot.	7
Synthesis of results	21	Describe test accuracy, including variability; if meta-analysis was done, include results and confidence intervals.	8
Additional analysis	23	Give results of additional analyses, if done (e.g., sensitivity or subgroup analyses, meta-regression; analysis of index test: failure rates, proportion of inconclusive results, adverse events).	9-11
**DISCUSSION**			
Summary of evidence	24	Summarize the main findings including the strength of evidence.	12

Limitations	25	Discuss limitations from included studies (e.g. risk of bias and concerns regarding applicability) and from the review process (e.g. incomplete retrieval of identified research).	12
Conclusions	26	Provide a general interpretation of the results in the context of other evidence. Discuss implications for future research and clinical practice (e.g. the intended use and clinical role of the index test).	13
**FUNDING**			
Funding	27	For the systematic review, describe the sources of funding and other support and the role of the funders.	Not applicable

Table S1: Preferred Reporting Items for Systematic Reviews and Meta-analyses of Diagnostic Test Accuracy Studies (PRISMA-DTA) Checklist

Table S2:

**Database**	**Index and keyword terms**
**PubMed**	(“artificial intelligence” [Mesh]) OR (“artificial intelligence” [Title/Abstract]) OR (“AI” [Title/Abstract]) OR (“machine learning” [Mesh]) OR (“machine learning” [Title/Abstract]) OR (“deep learning” [Mesh]) OR (“deep learning” [Title/Abstract]) OR (“algorithm” [Title/Abstract]) OR (“neural network” [Title/Abstract]) OR (“computer intelligence” [Title/Abstract])) AND ((“computed tomography” [Mesh]) OR (“computed tomography” [Title/Abstract]) OR (“magnetic resonance imaging” [Mesh]) OR (“magnetic resonance imaging” [Title/Abstract]) OR (“MR imaging” [Title/Abstract]) OR (“ultrasonography” [Mesh]) OR (“ultrasonography” [Title/Abstract]) OR (“ultrasound” [Title/Abstract]) OR (“US” [Title/Abstract]) OR (“shear wave elastography” [Title/Abstract]) OR (“transient elastography” [Title/Abstract])) AND ((“liver fibrosis” [Title/Abstract]) OR (“Metabolic Dysfunction-Associated Steatotic Liver Disease” [Title/Abstract]) OR (“MASLD” [Title/Abstract]) OR (“Non-alcoholic Fatty Liver Disease” [Title/Abstract]) OR (“NAFLD” [Title/Abstract])) AND (“diagnostic accuracy” [Title/Abstract] OR “sensitivity” [Title/Abstract] OR “specificity” [Title/Abstract] OR “AUC” [Title/Abstract] OR “accuracy” [Title/Abstract])
EMBASE	('artificial intelligence':ab,ti OR 'machine learning':ab,ti OR 'deep learning':ab,ti OR 'neural networks':ab,ti OR 'algorithm':ab,ti OR 'computer intelligence':ab,ti) AND ('computed tomography':ab,ti OR 'magnetic resonance imaging':ab,ti OR 'MR imaging':ab,ti OR 'ultrasonography':ab,ti OR 'ultrasound':ab,ti OR 'US':ab,ti OR 'shear wave elastography':ab,ti OR 'transient elastography':ab,ti) AND ('liver fibrosis':ab,ti OR 'Metabolic Dysfunction-Associated Steatotic Liver Disease':ab,ti OR 'MASLD':ab,ti OR 'Non-alcoholic Fatty Liver Disease':ab,ti OR 'NAFLD':ab,ti) AND ('diagnostic accuracy':ab,ti OR 'sensitivity':ab,ti OR 'specificity':ab,ti OR 'auc':ab,ti OR 'accuracy':ab,ti)
The Cochrane Library (CENTRAL-trials only)	(“artificial intelligence” OR “machine learning” OR “deep learning” OR “algorithm” OR “neural networks” OR “computer intelligence” OR “computed tomography” OR “magnetic resonance imaging” OR “MR imaging” OR “ultrasonography” OR “ultrasound” OR “US” OR “shear wave elastography” OR “transient elastography”) AND (“liver fibrosis” OR “Metabolic Dysfunction-Associated Steatotic Liver Disease” OR “MASLD” OR “Non-alcoholic Fatty Liver Disease” OR “NAFLD”) AND (“diagnostic accuracy” OR “specificity” OR “sensitivity” OR “AUC”):ab,ti,kw
CINAHL	(TI (diagnostic accuracy OR sensitivity OR specificity OR AUC) OR AB (diagnostic accuracy OR sensitivity OR specificity OR AUC)) AND (TI (liver fibrosis OR Metabolic Dysfunction-Associated Steatotic Liver Disease OR MASLD OR Non-alcoholic Fatty Liver Disease OR NAFLD)) AND (TI (artificial intelligence OR machine learning OR deep learning OR computer intelligence OR algorithm OR neural network) OR

	AB (artificial intelligence OR machine learning OR deep learning OR computer intelligence OR algorithm OR neural network OR computed tomography OR magnetic resonance imaging OR MR imaging OR ultrasonography OR ultrasound OR shear wave elastography OR transient elastography) OR MH artificial intelligence)
Scopus	TITLE-ABS (“artificial intelligence” OR “machine learning” OR “deep learning” OR “algorithm” OR “neural networks” OR “computer intelligence”) AND TITLE-ABS (“computed tomography” OR “magnetic resonance imaging” OR “MR imaging” OR “ultrasonography” OR “ultrasound” OR “US” OR “shear wave elastography” OR “transient elastography”) AND TITLE-ABS (“liver fibrosis” OR “Metabolic Dysfunction-Associated Steatotic Liver Disease” OR “MASLD” OR “Non-alcoholic Fatty Liver Disease” OR “NAFLD”) AND TITLE-ABS (“diagnostic accuracy” OR “specificity” OR “sensitivity” OR “AUC”)
Google Scholar	(TI=(“artificial intelligence” OR “machine learning” OR “deep learning” OR “algorithm” OR “neural networks” OR “computer intelligence” OR “computed tomography” OR “magnetic resonance imaging” OR “MR imaging” OR “ultrasonography” OR “ultrasound” OR “shear wave elastography” OR “transient elastography”)) AND (TI=(“diagnostic accuracy” OR “specificity” OR “sensitivity” OR “AUC”) OR AB=(“diagnostic accuracy” OR “specificity” OR “sensitivity” OR “AUC”)) AND (TI=(“liver fibrosis” OR “Metabolic Dysfunction-Associated Steatotic Liver Disease” OR “MASLD” OR “Non-alcoholic Fatty Liver Disease” OR “NAFLD”) OR AB=(“liver fibrosis” OR “Metabolic Dysfunction-Associated Steatotic Liver Disease” OR “MASLD” OR “Non-alcoholic Fatty Liver Disease” OR “NAFLD”))
Web of Sciences	(ab,ti(“artificial intelligence”) OR ab,ti(“machine learning”) OR ab,ti(“deep learning”) OR ab,ti(“algorithm”) OR ab,ti(“neural networks”) OR ab,ti(“computer intelligence”)) AND (ab,ti(“computed tomography”) OR ab,ti(“ultrasound”) OR ab,ti(“magnetic resonance imaging”) OR ab,ti(“MR imaging”) OR ab,ti(“ultrasonography”) OR ab,ti(“US”) OR ab,ti(“shear wave elastography”) OR ab,ti(“transient elastography”)) AND (ab,ti(“liver fibrosis”) OR ab,ti(“Metabolic Dysfunction-Associated Steatotic Liver Disease”) OR ab,ti(“MASLD”) OR ab,ti(“Non-alcoholic Fatty Liver Disease”) OR ab,ti(“NAFLD”)) AND (ab,ti(“diagnostic accuracy”) OR ab,ti(“specificity”) OR ab,ti(“sensitivity”) OR ab,ti(“AUC”))

Table S2: Search Strategy.

Table S3

DOMAIN 1: PATIENT SELECTION

A. Risk of Bias

Describe methods of patient selection:

- Was a consecutive or random sample of patients enrolled? Yes/No/Unclear
- Was a case-control design avoided? Yes/No/Unclear
- Did the study avoid inappropriate exclusions? Yes/No/Unclear

Could the selection of patients have introduced bias? RISK: LOW/HIGH/UNCLEAR

B. Concerns regarding applicability

Describe included patients (prior testing, presentation, intended use of index test and setting):

Is there concern that the included patients do not match CONCERN:

LOW/HIGH/UNCLEAR the review question?

DOMAIN 2: INDEX TEST(S)

If more than one index test was used, please complete for each test.

A. Risk of Bias

Describe the index test and how it was conducted and interpreted:

- Were the index test results interpreted without Yes/No/Unclear knowledge of the results of the reference standard?
- If a threshold was used, was it pre-specified? Yes/No/Unclear **Could the conduct or interpretation of the index test RISK: LOW /HIGH/UNCLEAR have introduced bias?**

B. Concerns regarding applicability

Is there concern that the index test, its conduct, or CONCERN: LOW /HIGH/UNCLEAR interpretation differ from the review question?

DOMAIN 3: REFERENCE STANDARD

A. Risk of Bias

Describe the reference standard and how it was conducted and interpreted:

- Is the reference standard likely to correctly classify the target Yes/No/Unclear condition?
- Were the reference standard results interpreted without Yes/No/Unclear knowledge of the results of the index test?

Could the reference standard, its conduct, or its RISK: LOW /HIGH/UNCLEAR interpretation have introduced bias?

B. Concerns regarding applicability

Is there concern that the target condition as defined by CONCERN: LOW /HIGH/UNCLEAR the reference standard does not match the review question?

DOMAIN 4: FLOW AND TIMING

A. Risk of Bias

Describe any patients who did not receive the index test(s) and/or reference standard or who were excluded from the 2x2 table (refer to flow diagram):

Describe the time interval and any interventions between index test(s) and reference standard:

- ❖ Was there an appropriate interval between index test(s) Yes/No/Unclear and reference standard?
- ❖ Did all patients receive a reference standard? Yes/No/Unclear
- ❖ Did patients receive the same reference standard? Yes/No/Unclear

- ❖ Were all patients included in the analysis? Yes/No/Unclear **Could the patient flow have introduced bias? RISK: LOW /HIGH/UNCLEAR**

Table S3: QUADAS-2

Table S4

Study (Year)	Imaging Modality	AI Model Type	Training Set Size	Validation/ Test Set	Validation Approach	Ground Truth Standard	Feature Selection Method
Ahmed et al. (2019)	MRI	SVM	ND	37 patients	Leave-one-out CV	Histology (biopsy-confirmed fibrosis)	Manual selection
Choi et al. (2018)	CT	CNN	ND	7461 patients	Independent test set	Liver biopsy	Deep feature learning
Han et al. (2020)	MRI	CNN	ND	204 patients	External validation	Proton density fat fraction (MRI-PDFF)	Automated feature extraction
Hectors et al. (2021)	MRI	CNN	ND	355 patients	Cross-validation	Histopathology	Feature engineering
Lee et al. (2020)	Ultrasonography	DCNN	13,608 images	3446 patients	External test set	Biopsy and elastography	VGG-Net based filters
Li et al. (2019)	Ultrasound	SVM	ND	144 patients	Split-sample validation	Histology	Feature ranking & elimination
Li et al. (2020)	CT	ResNet	ND	347 patients	Independent testing	Biopsy	ResNet-50 pretrained weights
Wang et al. (2019)	SWE	CNN	1990 images	398 patients	Internal validation	SWE metrics & clinical scores	2D-SWE wavefront modeling
Yasaka et al.	MRI	CNN	144,180	634 patients	Independent test	Biopsy	3-layer CNN

(2017)			images		set		architecture
Yasaka et al. (2018)	CT	CNN	496 images	286 patients	Hold-out test	Histology	Batch normalization
Yin et al. (2021)	CT	CNN	ND	252 patients	Independent test	Histology + imaging consensus	Grad-CAM for interpretability
Zamanian et al. (2021)	Ultrasonography	SVM	ND	55 patients	Cross-validation	NAFLD diagnosis (clinical + imaging)	Kernel-based distance metrics
Zhang et al. (2012)	Duplex US	ANN	ND	239 patients	Validation set only	Fibrosis staging	3-layer neural network
Zhang et al. (2024)	Transient Elastography	RF	ND	916 patients	Bootstrap validation	TE stiffness values	Majority voting strategy
Zhu et al. (2021)	MRI	CNN	ND	123 patients	External testing	Biopsy-confirmed fibrosis	Custom CNN with 5 layers

Table S4: Methodological Characteristics of Included Studies on AI Applications in Abdominal Imaging for Liver Disease. Methodological characteristics of studies utilizing AI in abdominal imaging for liver disease. It includes details on imaging modalities, AI model types, training and validation set sizes, feature selection methods, and the ground truth standards used in each study. Abbreviations: CNN – Convolutional Neural Network; SVM – Support Vector Machine; DCNN – Deep Convolutional Neural Network; ANN – Artificial Neural Network; RF – Random Forest; SWE – Shear Wave Elastography; TE – Transient Elastography; CV – Cross-Validation; ND – Not Disclosed.

Table S5

Study (Year)	Clinical Use Case	Strengths of AI Application	Limitations Noted by Authors	Translational Readiness
Ahmed et al. (2019)	Fibrosis staging (MRI)	High specificity; leave-one-out validation	Small sample size; single-center data	Low
Choi et al. (2018)	CT-based liver fibrosis detection	Large dataset; robust CNN architecture	Limited generalizability to non-Asian cohorts	Moderate
Han et al. (2020)	NAFLD quantification	Fat fraction estimation with imaging biomarkers	Requires advanced MRI techniques	Moderate
Hectors et al. (2021)	Fibrosis grading (MRI)	Integration of automated pre-processing	Computational complexity	Moderate
Lee et al. (2020)	Ultrasound fibrosis analysis	VGG-Net accuracy; external test validation	Operator-dependent imaging	Moderate
Li et al. (2019)	US fibrosis staging	Feature reduction to prevent overfitting	Modest sample size; manual inputs	Low
Li et al. (2020)	CT fibrosis analysis	Deep ResNet model; clear model training pipeline	No real-time clinical interface	Moderate
Wang et al. (2019)	SWE-based fibrosis detection	Non-invasive SWE integration; CNN performance	Lack of external validation	Moderate
Yasaka et al. (2017)	MRI fibrosis classification	High image volume; consistent performance	Requires specialized MRI protocols	High
Yasaka et al. (2018)	CT fibrosis staging	Simple CNN implementation; Python-based	Retrospective design	Moderate
Yin et al. (2021)	CT fibrosis staging	Visual interpretation via	High resource demand	Moderate

		Grad-CAM		
Zamanian et al. (2021)	US-based NAFLD detection	Good sensitivity; explainable kernel model	Limited population diversity	Low
Zhang et al. (2012)	Duplex US fibrosis classification	ANN efficiency for binary classification	Technology outdated	Low
Zhang et al. (2024)	TE-based fibrosis staging	RF ensemble learning with aggregation	Dependent on elastography device quality	Moderate
Zhu et al. (2021)	MRI fibrosis assessment	Deep CNN architecture with multi-layer learning	Small cohort; no validation set	Low

Table S5: Clinical Implications and Limitations of AI Use in Liver Imaging Across Studies. Clinical implications and limitations of AI applications in liver imaging. This table summarizes the clinical use cases, strengths, limitations, and translational readiness of AI models as reported in the studies, highlighting the potential for clinical implementation. Note: Translational readiness was qualitatively ranked as *Low*, *Moderate*, or *High* based on study design robustness, sample size, external validation, and potential for integration into clinical workflows.

Table S6

Study	Risk of bias				Applicability concerns		
	Patient selection	Index test	Reference standard	Flow and timing	Patient selection	Index test	Reference standard
Ahmed et al., 2019	☹	☹	☺	☺	☹	☹	☺
Choi et al., 2018	☺	☹	☺	☺	☺	☹	☺
Han et al., 2020	☺	☺	☺	☺	☺	☺	☺
Hectors et al., 2021	☹	☺	☺	☺	☺	☺	☺
Lee et al., 2020	☺	☹	☺	☺	☺	☹	☺
Li et al., 2019	☺	☹	☺	☺	☺	☺	☺
Li et al., 2020	☹	☺	☺	☺	☺	☺	☺
Wang et al., 2019	☺	☹	☺	☺	☺	☹	☺
Yasaka et al., 2017	☺	☺	☺	☺	☺	☺	☺
Yasaka et al., 2018	☺	☹	☺	☺	☺	☺	☺
Yin et al., 2021	☺	☹	☺	☺	☺	☹	☺
Zamanian et al., 2021	☺	☹	☺	☺	☺	☹	☺
Zhang et al., 2012	☺	☺	☺	☺	☺	☺	☺
Zhang et al., 2024	☺	☺	☺	☺	☺	☺	☺
Zhu et al., 2021	☹	☹	☺	☺	☹	☹	☺

☺ Low Risk ☹ High Risk ? Unclear Risk

Table S6: Tabular presentation for QUADAS-2 results for individual studies.

Table S7

Study	Risk of bias				Applicability Concerns		
	Patient selection	Index test	Reference standard	Flow and timing	Patient selection	Index test	Reference standard
Ahmed et al., 2019	X	X	✓	✓	X	X	✓
Choi et al., 2018	✓	X	✓	✓	✓	X	✓
Han et al., 2020	✓	✓	✓	✓	✓	✓	✓
Hectors et al., 2021	X	✓	✓	✓	✓	✓	✓
Lee et al., 2020	✓	X	✓	✓	✓	X	✓
Li et al., 2019	✓	X	✓	✓	✓	✓	✓
Li et al., 2020	X	✓	✓	✓	✓	✓	✓
Wang et al., 2019	✓	X	✓	✓	✓	X	✓
Yasaka et al., 2017	✓	✓	✓	✓	✓	✓	✓
Yasaka et al., 2018	✓	✓	✓	✓	✓	✓	✓
Yin et al., 2021	✓	X	✓	✓	✓	✓	✓
Zamanian et al., 2021	✓	X	✓	✓	✓	X	✓
Zhang et al., 2012	✓	✓	✓	✓	✓	✓	✓
Zhang et al., 2024	✓	✓	✓	✓	✓	✓	✓
Zhu et al., 2021	X	X	✓	✓	X	X	✓

✓: low risk, X: high risk, ?: unclear risk

Table S7: Overall agreement between reviewers for each of the QUADAS domains

Table S8

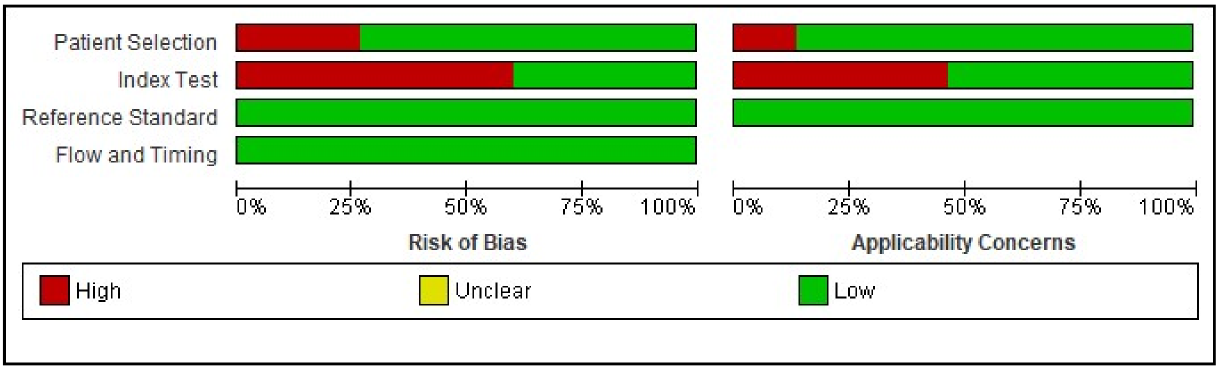

Table S8: Risk of bias and applicability concerns graph: authors' judgments as percentages across studies. High applicability concerns in 2 studies (patient selection) and 7 studies (index tests) suggest limitations in study design, highlighting the need for more rigorous designs in future AI research.

Table S9

Study	PLR (95% CI)	NLR (95% CI)	DOR (95% CI)
[24]	11.71 (5.70–24.07)	0.05 (0.02–0.12)	188.90 (59.96–595.14)
[25]	8.41 (4.90–14.45)	0.09 (0.05–0.16)	92.98 (37.24–232.22)
[26]	6.88 (3.88–12.22)	0.14 (0.09–0.23)	49.78 (23.91–103.61)
[27]	5.56 (3.53–8.77)	0.16 (0.10–0.26)	33.68 (17.23–65.83)
[28]	5.20 (3.27–8.27)	0.19 (0.12–0.30)	27.76 (13.95–55.24)
[29]	4.69 (2.89–7.62)	0.21 (0.13–0.34)	22.43 (10.67–47.14)
[30]	4.32 (2.74–6.81)	0.23 (0.15–0.36)	19.10 (9.17–39.75)
[31]	3.70 (2.40–5.71)	0.26 (0.17–0.39)	15.06 (7.43–30.53)
[32]	3.19 (2.06–4.95)	0.29 (0.19–0.44)	11.18 (5.55–22.53)
[33]	2.68 (1.75–4.10)	0.32 (0.21–0.48)	8.40 (4.22–16.71)
[34]	2.32 (1.57–3.42)	0.34 (0.23–0.50)	6.84 (3.46–13.52)
[35]	2.02 (1.57–2.60)	0.35 (0.24–0.51)	6.73 (3.61–12.54)

Table S9: Detailed Diagnostic Performance Metrics (PLR, NLR, and DOR) of AI-Based Abdominal Imaging for Liver Fibrosis Across Included Studies. Detailed diagnostic performance metrics — positive likelihood ratio (PLR), negative likelihood ratio (NLR), and diagnostic odds ratio (DOR) — for AI-based abdominal imaging in liver fibrosis detection across 12 individual studies. The wide ranges highlight variability in diagnostic strength, supporting the meta-analysis findings of significant heterogeneity.

Table S10

Study	DOR (95% CI)
[36]	19.58 (8.55–44.85)
[37]	305.08 (13.53–6877.82)
[38]	5950.82 (300.03–118029.49)

Table S10: Detailed Diagnostic Odds Ratio (DOR) Distribution for AI-Based Abdominal Imaging in MASLD Across Included Studies. Diagnostic odds ratios (DOR) for AI-assisted abdominal imaging in diagnosing MASLD across three included studies. The DOR values illustrate substantial variability, with one study reporting an exceptionally high diagnostic capacity, reinforcing the observed heterogeneity in the meta-analysis.

Figure S1

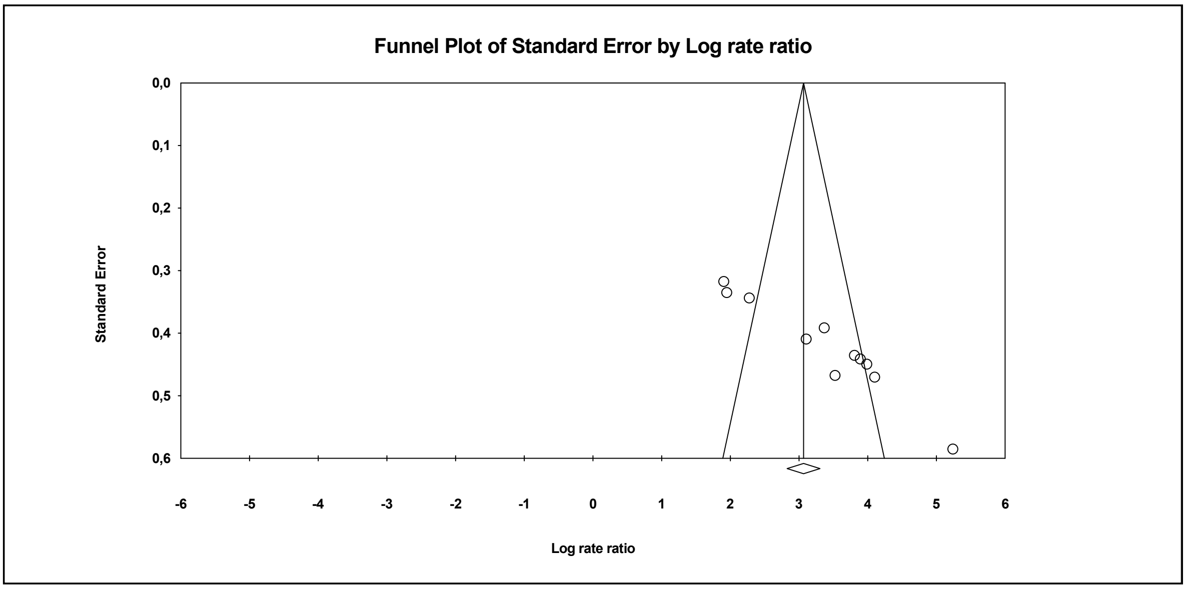

Figure S1. Funnel plot of pooled DOR for AI-based abdominal imaging in diagnosing liver fibrosis. Each circle represents one of the 12 studies included. The x-axis shows the log rate ratio; the y-axis shows the standard error. The vertical line marks the fixed-effects summary, with 95% CI bounds shown by the sloped lines. Egger's test indicates significant publication bias ($p = 0.000$), suggesting a tendency to publish positive results, underscoring the need for balanced reporting.
